# Understanding the Chemical Composition and Biological Activities of Different Extracts of *Secamone afzelii* Leaves: A Potential Source of Bioactive Compounds for the Food Industry

**DOI:** 10.3390/molecules28093678

**Published:** 2023-04-24

**Authors:** Kouadio I. Sinan, Sakina Yagi, Eulogio J. Llorent-Martínez, Antonio Ruiz-Medina, Ana I. Gordo-Moreno, Azzurra Stefanucci, Adriano Mollica, Kouadio Bene, Gokhan Zengin

**Affiliations:** 1 Department of Biology, Science Faculty, Selcuk University, Konya 42130, Turkey; 2 Department of Botany, Faculty of Science, University of Khartoum, Khartoum 11115, Sudan; 3 Department of Physical and Analytical Chemistry, University of Jaén, Campus Las Lagunillas S/N, 23071 Jaén, Spain; 4 Department of Pharmacy, University “G. d’Annunzio” of Chieti-Pescara, 66100 Chieti, Italy; 5 Laboratoire de Botanique et Phytothérapie, Unité de Formation et de Recherche Sciences de la Nature, Université Nangui Abrogoua, Abidjan 02 BP 801, Côte d’Ivoire

**Keywords:** *Secamone afzelii*, antioxidant, enzyme inhibition, chemical profile, extraction solvent, functional food

## Abstract

*Secamone afzelii* (Roem. & Schult.) K. Schum (family Asclepiadaceae) is a creeping woody climber used to treat ailments in many traditional medicine systems. The present study aims to examine the antioxidant and enzyme inhibition activities of *S. afzelii* leaf using different compositions of methanol–water mixture as an extraction solvent. The extracts were characterized by HPLC-ESI-MS^n^ in terms of chemical compounds. The in silico results show that compound **23** (quercitrin) has the higher docking scores among the selected substances and the MD simulation revealed that the interactions with the enzymatic pocket are stable over the simulation time and strongly involve the tyrosinase catalytic Cu atoms. All together the results showed that both 80% and 100% methanolic extracts contained significantly (*p* < 0.05) the highest total phenolics content while the highest content of total flavonoids was significantly (*p* < 0.05) extracted by 100% methanol. About 26 compounds were tentatively identified by HPLC-ESI-MS^n^ and 6 of them were quantified using standards. Results showed that the extracts were rich in flavonoids with a relatively high abundance of two kaempferol glycosides comprising 60% of quantified compounds. The 100% and 80% methanol extracts recorded significantly (*p* < 0.05) the highest total antioxidant, DPPH and ABTS activity as well as tyrosinase and ⍺-amylase inhibitory activities. The best significant (*p* < 0.05) cholinesterase inhibitory activity and reducing capacity of Fe^+++^ and Cu^++^ was recorded from the 80% methanolic extract while 100% ethanolic extract gave the highest significant (*p* < 0.05) butyrylcholinesterase inhibitory activity. The best glucosidase activity was observed in the 50% and 80% methanolic extracts. Although the water extract displayed the least total phenolics and flavonoids content and consequently the lowest antioxidant and enzyme inhibition activity, it displayed significantly (*p* < 0.05) the highest chelating power. In conclusion, these results demonstrated the richness of *S. afzelii* leaf as a potential source of bioactive compounds for the food industry, for the preparation of food supplements and functional foods.

## 1. Introduction

Secondary metabolites with their limitless structural diversity and biological activity afford huge possibilities for plant-based biomolecules for drug discovery [1]. Plants are widely considered powerful sources of specialized metabolites with many beneficial effects on human health, such as antioxidative, anti-inflammatory, and cardioprotective properties, thus preventing obesity and regulating diabetes, among others [2]. Accordingly, there has been a consistent interest in identifying new sources rich in bioactive components as well as new approaches for their preparation [3]. Finding novel sources of natural bioactive compounds is currently an interesting approach for the design of novel pharmaceuticals, food supplements, and functional foods.

The nature and content of biomolecules are highly influenced by extraction solvents and techniques employed [4]. Plants contain complex mixtures of many metabolites varied in their polarity, so to obtain high functional properties of the extract required, it is important to select the efficient solvent extraction and procedure. Polar solvents and aqueous mixtures containing ethanol, methanol, acetone, and ethyl acetate are found to be appropriate for the extraction of phenolics compounds with high antioxidant activity. For example, 100% ethanolic extract from *Limnophila aromatica* displayed better total phenolic content and antioxidant activity than 75% and 50% ethanolic extracts [5]. Extraction with aqueous ethanol (70%) and methanol (70%) revealed the highest antioxidant activity of aerial parts of some root vegetables such as *Raphanus sativus, Beta vulgaris,* and *Daucus carota* [6]. However, extraction with water is advantageous as it is non-toxic and environmentally friendly and thus considered the greenest solvent [7].

*Secamone afzelii* (Roem. & Schult.) K. Schum (family Asclepiadaceae) is a creeping woody climber widely distributed in Asia and Africa. It is used commonly in many traditional medicine systems to treat ailments such as digestive system problems, cough, gonorrhea, diabetes, kidney problems, backache, spinal disease, catarrhal conditions, and reproductive abnormalities [8,9,10]. In addition, in many indigenous African societies, a number of herbs and vegetables, among them leaves of *S. afzelii*, are incorporated in the diet of pregnant and lactating mothers for maintenance of well-being, prevention of anemia, and stimulation of milk production [11]. The plant has been found to possess antioxidant [9,12], antimicrobial [13], insecticidal [14], and anti-inflammatory [15] activities. Although *S. afzelii* was shown to contain phenolics, alkaloids, coumarines, tannins, cardiac glycosides, and saponins ([8,16], few reports identified the chemical constituents or presented the chemical profile of different parts of the plant. The most detailed work was performed by Magid, et al. [17] who isolated and identified two new diglycoside flavonoids besides nine other known flavonoids from the aerial parts.

Considering all the aforementioned works, this work aims to evaluate the antioxidant and enzyme inhibition activities of *S. afzelii* leaves and to highlight the effect of solvent extraction by using different compositions of methanol–water. Furthermore, the various individual secondary metabolites found in different extracts were tentatively identified by HPLC-ESI-MS^n^. The results generated from this work provide for the first time an overview of the secondary metabolites present in *S. afzelii* leaf, as well as delineate its antioxidative and enzyme inhibition potential, and makes this plant a candidate for the obtention of bioactive compounds to develop novel food supplements of functional foods.

## 2. Results and Discussion

### 2.1. Total Phenolics and Flavonoids Content

The total phenolics and flavonoids contents in the 50%, 80%, and 100% methanolic and water extracts of *S. afzelii* leaves were determined and the results are depicted in Table 1. Both 80% and 100% methanolic extracts contained significantly (*p* < 0.05) the highest total phenolics content. These values were higher than that obtained for *S. afzelii* plant grown in Ghana, where the total phenolic content of the leaf was 56.86 mg tannic acid equivalent/g [15]. Furthermore, the 100% methanolic extract displayed significantly (*p* < 0.05) the highest total flavonoids content followed by the 80% methanolic extracts. The 50% methanolic extract followed by the water extract had the least total phenolics and flavonoids contents. It was observed that water and organic solvents with a high proportion of water extracted other molecules such as carbohydrates and terpenes and thus had relatively lower phenol concentrations [5].

### 2.2. HPLC-ESI-MS^n^ Analysis

**Figure 1:**
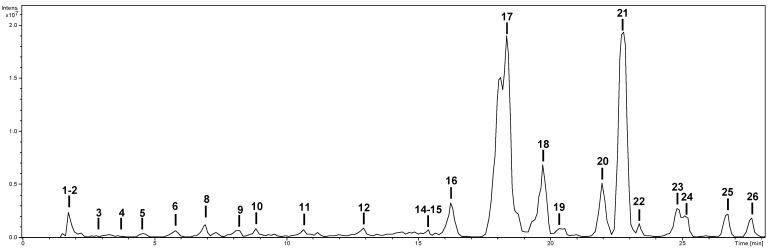
Base peak chromatogram of the methanol extract of *S. afzelii*.

The characterization of the phytochemicals was carried out by HPLC-ESI-MS^n^. Identification was performed using analytical standards—citric acid, caffeic acid, protocatechuic acid, procyanidin B1, kaempferol, luteolin, quercetin, rutin, and vicenin-2—as well as bibliographic information. As an example, the base peak chromatogram of the methanolic extract is shown in Figure 1. The characterization of the compounds in all extracts is shown in Table 2. Compounds were numbered according to their elution order, keeping the same numbering in all extracts. A brief explanation of the characterization of the compounds not identified by analytical standards follows.

Compound **1** was tentatively characterized as a diglucoside (HCl adduct) due to the neutral loss of 162 Da (341→179) and the characteristic fragments of hexoside moieties (*m*/*z* 179, 161, 143, 131, and 113) [18].

Compounds **2** and **3** exhibited the same fragmentation pattern, corresponding to (iso)citric acid. The distinction between both isomers was performed by analyzing an analytical standard of citric acid.

Compound **4** displayed the neutral loss of 162 Da to yield dihydroxybenzoic acid at *m*/*z* 153 (fragment ion at *m*/*z* 109), so it was tentatively characterized as dihydroxybenzoic acid-*O*-hexoside. An analytical standard of protocatechuic acid was used to confirm the fragmentation of the dihydroxybenzoic acid.

Compound **5** displayed the base peak at *m*/*z* 179 (main fragment at *m*/*z* 135), which corresponded to caffeic acid, so it was tentatively characterized as a derivative.

Compound **6** was identified as trytophan by comparison of the mass spectrum with bibliographic information [19].

Compounds **8**, **9**, and **15** were characterized as procyanidin dimers by using an analytical standard of procyanidin B1.

Compound **11** was characterized as the formate adduct of roseoside (vomifoliol glucoside or drovomifoliol-*O*-β-d-glucopyranoside) [20].

For the identification of flavonoid glycosides, the neutral losses of 132, 146, 162, and 308 Da indicated the presence of pentoside, deoxyhexoside, hexoside, and rutinoside moieties, respectively. The aglycones were identified by the use of analytical standards. Hence, quercetin was identified at *m*/*z* 301 (fragment ions at *m*/*z* 179 and 151), kaempferol at *m*/*z* 285 (fragment ion at *m*/*z* 255) and luteolin at *m*/*z* 285 (fragment ion at *m*/*z* 243, which is absent in kaempferol). In all cases, the flavonoid glycosides were *O*-glycosilated, except compound **13**, which was *C*- and *O*- glycosilated.

The assignment of the exact isomers to compounds **17**, **20**, **21**, **23**, and **24** (Table 2) was based on the most common positions for the moieties observed in kaempferol, luteolin, and quercetin. To decide the most probable exact isomers (the ones used for docking experiments), we perform a thorough search in databases, scientific articles, and analytical standards available in commercial manufacturers.

### 2.3. Quantification of Phytochemicals

The quantitation of flavonoids is shown in Table 3. It can be observed that the total concentration of glycosides was similar in all extracts except in the aqueous extract, in which the recovery was much lower. The most abundant compounds were two kaempferol glycosides (compounds **17** and **21**). In fact, many studies have revealed that extraction with methanol or hydromethanol recovered the highest yield of phenols and flavonoids [21,22,23] while water was less effective [24].

After performing the quantitation of the most abundant compounds, we also calculated the relative contribution of all compounds using the method of area normalization. Peak areas of each compound were obtained using the precursor ion, [M-H]^-^, (extracted ion chromatograms). Then, the relative contribution (in percentage) of each compound was calculated and the heat map (the darker the color, the higher the abundance) was constructed (Table 4). It can be observed that these data are in agreement with the quantification (Table 3). Similarly, these compounds were also reported in previous studies [17,25]. The most abundant compounds were compounds **17** and **21**, which accounted for more than 60% of the extracted compounds.

### 2.4. Antioxidant Capacity

**Figure 2:**
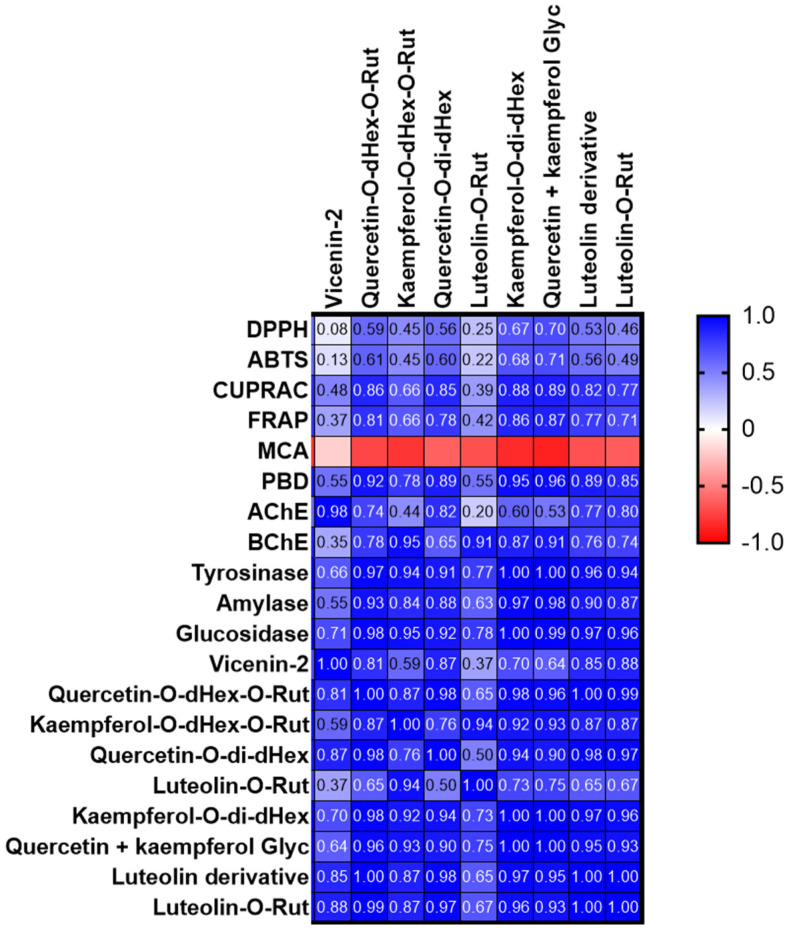
Pearson’s correlation between quantified compounds and antioxidant/enzyme inhibitory assays (MCA: metal chelating assay; PBD: phosphomolybdenum assay).

The antioxidant activity of *S. afzelii* leaves and the effect of extraction solvent were examined by testing the capacity of extracts to scavenge the DPPH and ATBS radicals, reduce ions and chelate the Fe ions. Results are presented in Table 5. Both the 100% and 80% methanolic extracts exerted significantly (*p* < 0.05) remarkable DPPH and ABTS scavenging activity. In fact, the 100% methanolic extract was highly active by 5.6 and 8.9-fold than the 50% methanolic and water extracts respectively in the DPPH assay while the 80% extract was highly active by 4.4 and 6.8- fold than the same two extracts in the ABTS assay. Variation in the capacity of extracts to scavenge the DPPH and ABTS radicals could be attributed to many factors such as stereoselectivity of the radicals or the solubility of the extracts in different testing systems might affect the capacity of extracts to react and quench different radicals [26,27]. Moreover, it was reported that in the DPPH experiment, the hydrogen supply capacity of a compound determines the scavenging effect of free radicals, while the scavenging effect of ABTS^·+^ is determined by the scavenging effect of proton free radicals by giving electrons [28]. Furthermore, the three methanolic extracts exhibited high reducing capacity with higher ability to reduce the Cu ions compared to the Fe ones and the 80% methanolic extract revealed significantly (*p* < 0.05) the highest values followed by the 100% and 50% methanolic extracts, respectively. The 100% and 80% methanolic extracts showed significantly (*p* < 0.05) the highest total antioxidant activity from the phosphomolybdenum assay. Interestingly, although the water extract revealed the least activity in the five precedent assays, it recorded significantly (*p* < 0.05) the best chelating capacity, 12.2 and 2.1 times greater than that exerted by the 100% and 80% methanolic extracts respectively. Moreover, the 50% methanolic extract showed the best activity among the other methanolic ones. The high antiradical and reducing capacity of 100% and 80% methanolic activity could be attributed to their highest total phenolics and flavonoids contents. However, it was found that the four extracts were characterized by a high accumulation of kaempferol glycosides (compounds **17** and **21**) with the highest amount recorded in the water extract. Thus, kaempferol derivatives might not be the main molecules responsible for the antioxidant activity of extracts. Jung, et al. [29] found that kaempferol glycosides varied in their antioxidant property according to the type and number of sugar moieties. Other compounds including quercetin derivatives (compounds **16**, **19**, and **23**) in addition to compounds **5**, **10**, **25**, and **26**, although present in relatively low abundance, could be more effective as individual antioxidant molecules than the two kaempferol glycosides or they may collectively exert synergistic effect that reflected in the high antioxidant of the methanolic extracts, particularly the 100% and 80% methanolic ones. Magid, Yao-Kouassi, Gossan, Mairot, and Voutquenne-Nazabadioko [17] isolated and identified 11 flavonoids from the aerial parts of *S. afzelii;* among them quercetin-3 -O-β-D-apiofuranosyl-(1→2)-α-L-rhamnopyranoside, quercitrin, and rutin exerted the highest anti-DPPH radicals activity. We also performed a Pearson correlation analysis (Figure 2) and some compounds were strongly correlated with antioxidant properties. In particular, compounds **21** and **23**/**24** mainly contributed to the observed antioxidant properties, with the exception of metal chelation. The low antioxidant activity of the water could possibly be due to its least content of total phenolics and flavonoids, in addition, some compounds known for their antioxidant activity such as catechin [30], caffeic acid derivative [31], and rutin [17] were not detected in the water extract. Moreover, it was observed that the water was the only solvent to recover the compound kaempferol-C-hexoside-O-deoxyhexoside besides a small amount of an unknown compound (peak 7, Table 2) which may in part be responsible for its remarkable chelating property.

### 2.5. Enzyme Inhibitory Effects

The water and three methanolic extracts were examined for their enzyme inhibition property against AChE, BChE, Tyr, ⍺-amylase, and α-glucosidase enzymes. Results are presented in Table 6. Only the 80% and 50% methanolic extracts possessed considerable anti-AChE activity with significant (*p* < 0.05) higher activity observed in the 80% methanolic extract. Furthermore, all three methanolic extracts revealed anti-BChE activity with the highest significant (*p* < 0.05) value recorded from the 100% methanolic extract. They also displayed considerable anti-Tyr property with the best significant (*p* < 0.05) activity obtained from both the 100% and 80% methanolic extracts. Upon testing the extracts for their capacity to inhibit the two enzymes associated with diabetes, it was observed that the three methanolic extracts moderately inhibited the ⍺-amylase enzyme but displayed remarkable activity against the ⍺-glucosidase enzyme with the highest significant (*p* < 0.05) activity obtained from both the 50% and 80% methanolic extracts. Complete inhibition of α-amylase affects the digestion and intestinal absorption of carbohydrate which might cause undesirable side effects and thus extracts exhibiting a low α-amylase and a high α-glucosidase inhibitory activity are preferred in the management of diabetes [32,33]. The water extract was either not active or showed weak activity against all the tested enzymes. Many bioactive compounds were previously evaluated for their enzyme-inhibition property. For example, quercetin and its glycosides are found to possess anti-AChE activity [34]. They also have demonstrated to exert anti-tyrosinase [35] and ⍺-glucosidase inhibition [36,37] activities with kaempferol glycosides. This fact was also supported by a correlation analysis (Figure 2) and these compounds positively correlated with enzyme inhibition effects. In particular, compound **21** and **23**/**24** correlated strongly with tyrosinase inhibitory effects (R > 0.9).

### 2.6. Molecular Dynamic Studies of Quercitrin with Tyrosinase

**Figure 3:**
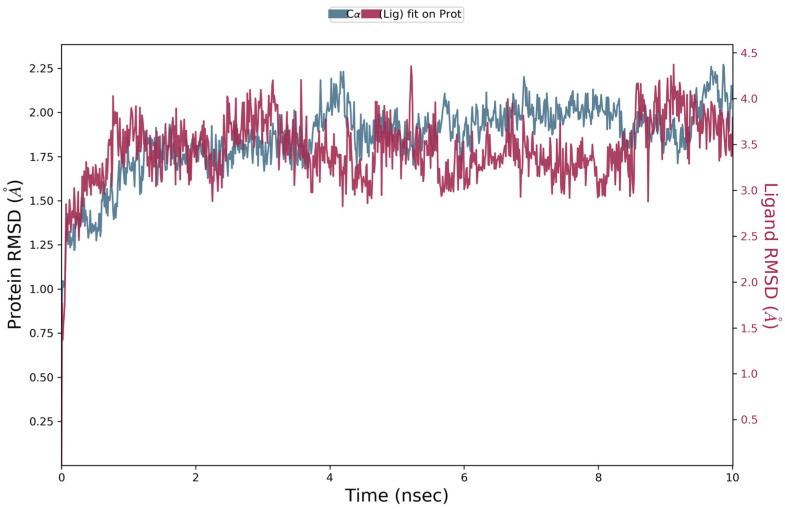
RMSD graphic of Cα of tyrosinase (blue) and quercitrin (Red) over a molecular dynamic simulation of 10 ns of tyrosinase docked to quercitrin.

**Figure 4:**
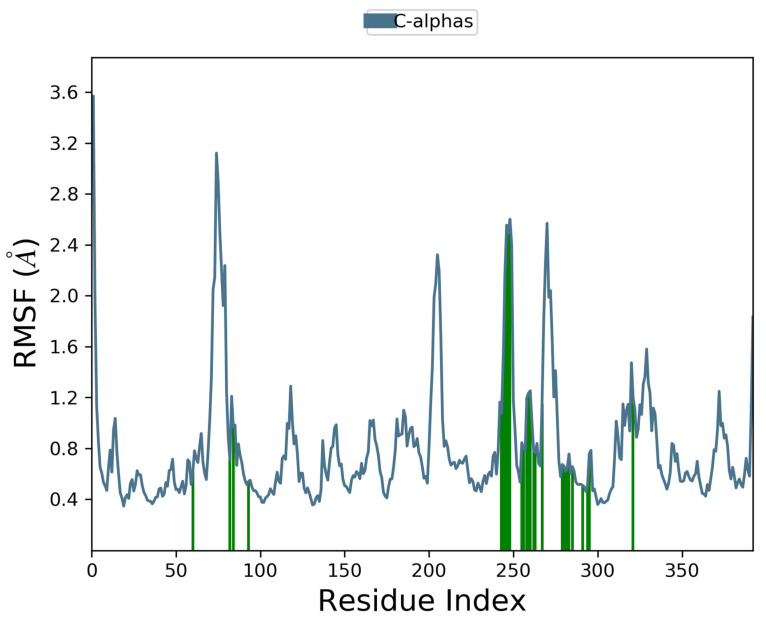
Interaction with the inhibitors has effect on the protein residues dynamic behavior of tyrosinase docked to quercitrin (**23**). In blue the residues involved in β-strands. The green lines highlight the residues of the enzyme in contact with the ligand ì.

**Figure 5:**
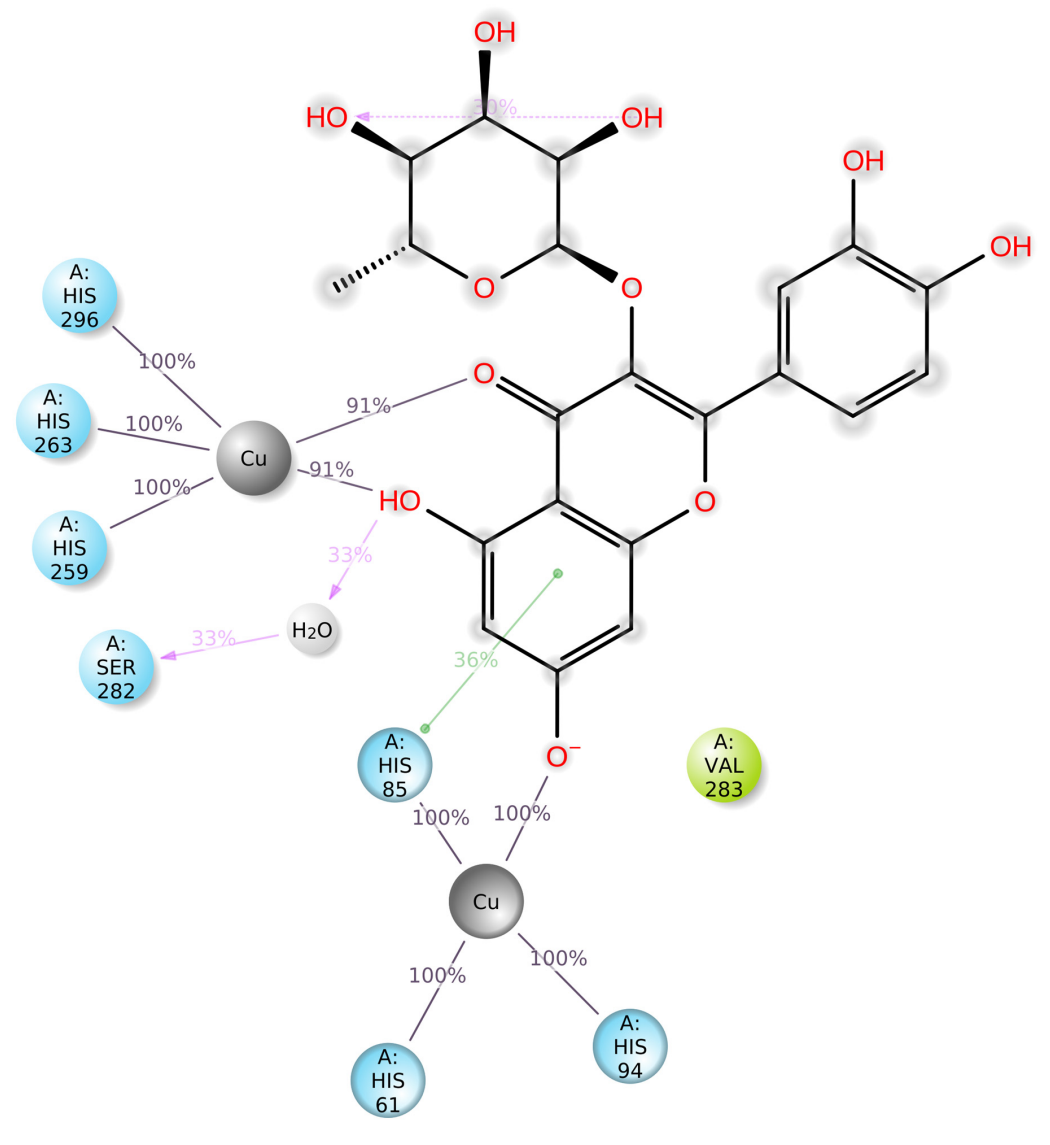
Graphical 2D representation of the stable interactions of quercitrin (compound **23**) docked to tyrosinase binding pocket during up to 90% of the MD simulation time.

Docking scores for selected compounds are reported in Table 7. We investigated the docking pattern of quercitrin (compound **23**) with tyrosinase using molecular docking and molecular dynamic. RMSD values of the Cα of tyrosinase and of the ligand docked to tyrosinase were calculated and presented in Figure 3 to study the interaction mode of quercitrin in the enzymatic cavity and the effect on the protein structure. The enzyme fluctuates around a maximum value of RMSD of around 2 Å, and the ligand fluctuates around 3.5 Å. The RMSF for the protein residues was also analyzed in order to evaluate the overall stability of the enzyme docked to 23, the RMSF graphical is reported in Figure 3. It can be argued that residue fluctuations of tyrosinase are elevated for the amino acid located between 60–90 and 240–280 positions, also another high peak is present normally at the residues located at the N and C terminal. However, the interactions with the ligand (highlighted in green in the graphic in Figure 4) fall for the majority in stable part of the protein. Next, we analyzed the total interactions maintained by the ligand during the simulation in terms of hydrogen bonds, π−π stacks, ionic interactions to Cu metals present in the protein a summary of this analysis is reported in Figure 5. The interactions to both Cu atoms have been maintained and coordinated by the π−π stacks to His244,259,263 which surround the metal atoms, and kept for over the 90% of the simulation whereas the hydrogen bonds to Ser282 and Gly281 are more labile and have been lost after few ns of simulation. These data suggest that the docking pose generated by Glide was further improved during the MD simulation indicating that this molecule has a high specificity for the enzymatic cavity of the protein. Our findings tend to corroborate with [38] who reported the anti-tyrosinase inhibitory potential of quercetin-derived substances, with computational models. This study offers insight into the inhibition activity found for rutin toward tyrosinase. Our molecular modeling approach demonstrated that rutin is capable of bonding to tyrosinase cavity by interacting with several amino acid side chains and toward one Cu atom in the enzymatic cavity. We also elucidated the binding mode of compound **23** namely quercitrin to tyrosinase using molecular docking and demonstrated that 23 was able to stably occupy the enzyme pocket.

## 3. Materials and Methods

### 3.1. Plant Materials and Preparation of Extracts

*Secamone afzelii* was collected in Côte d’Ivoire (Agboville, Region of Agnéby-Tiassa) in 2019 (at flowering season). Taxonomical identification was performed by a botanist (Dr. Kouadio Bene). The plant materials were cleaned thoroughly by washing them with tap water and rinsing them with distilled water to remove soil and contaminants. The leaves were then separated and dried for 10 days in a well-ventilated (humidity: 10–12%) and shaded environment at room temperature. The dried materials were ground into powder (particle size: 2 mm) using a Retsch SM-200 laboratory mill and extracted within the same week. The powdered plant material was stored in a cool, dark, and well-ventilated area at around 20 °C.

We used water and hydroalcholic extracts (50%, 80%, and 100% methanol) in the preparation of plant extracts. The ultrasound-assisted method was chosen and 5 g of plant material was mixed with 100 mL of these solvents at room temperature for 30 min in a sonication bath (Daihan, WUC-D10H, Wonju-si, Korea, ultrasonic density: 65 W/L). The mixtures were then filtered with Whatman 1 filter paper, and the solvents were removed with a rotary evaporator. Regarding water extract, the extracts were also filtered and lyophilized for 48 h. All extracts were kept at 4 °C until analysis.

### 3.2. Chromatographic Analysis

Chromatographic analyses were performed with an Agilent Series 1100 HPLC system with a G1315B diode array detector (Agilent Technologies) and an ion trap mass spectrometer (Esquire 6000, Bruker Daltonics) with an electrospray interface operating in negative ion mode. Separation was performed in a Luna Omega Polar C_18_ analytical column (150 × 3.0 mm; 5 µm particle size) with a Polar C_18_ Security Guard cartridge (4 × 3.0 mm), both purchased from Phenomenex. Detailed chromatographic conditions are available in [39].

The most abundant compounds (flavonoids) were quantified by UV signal at 350 nm and the following analytical standards: vicenin-2, kaempferol, luteolin, and quercetin. Calibration graphs were constructed in the 0.5–100 mg L^−1^ range. Peak areas at 350 nm were plotted against analyte concentration. Each analytical standard was used to quantify the corresponding compound or compounds of the same chemical family. Detection limits (3σ criterion) were 0.1–0.2 mg L^−1^. Repeatability (n = 10) and intermediate precision (n = 9, three consecutive days) were lower than 4 and 8%, respectively. The robustness of the chromatographic method was evaluated by recording analyte signals at ±2 nm of the optimum wavelength and by slightly varying the percentage of the mobile phase (2% changes), observing variations lower than 5% for all the analytes concerning the optimum conditions.

### 3.3. Determination of Total Phenolic, Flavonoid and Antioxidant, and Enzyme Inhibitory Effects

Total phenolic content (TPC), total flavonoid content (TFC), DPPH radical scavenging, ABTS radical scavenging, cupric reducing antioxidant capacity (CUPRAC), ferric reducing antioxidant power (FRAP), metal chelating activity (MCA), phosphomolybdenum (PBD), inhibition of acetylcholinesterase (AChE), butyrylcholinesterase (BChE), tyrosinase, amylase, and glucosidase assays were performed as previously described [40,41]. Gallic acid and rutin were used as standard compounds to evaluate the levels of total phenolic and flavonoid content in the extracts. Trolox (for DPPH, ABTS, CUPRAC, FRAP, and PBD) and EDTA (for metal chelating assay) were used as standard compounds in the antioxidant assays. Galanthamine (for AChE and BChE), kojic acid (for tyrosinase), and acarbose (for amylase and glucosidase) were standard enzyme inhibitors in the enzyme inhibition assays. Each sample was processed in triplicate.

### 3.4. In Silico Experiments

#### 3.4.1. Enzyme Preparation

The enzyme three-dimensional structure tyrosinase was downloaded in raw PDB format from the free available Protein Data Bank (2Y9X) [42]. The co-crystallized tropolone-enzyme was made suitable for computational calculations using PrepWizard module of Maestro 2021 [43]. The crystal structure was prepared by removing water molecules, salts, and neutralization was carried out at physiological pH by PropKa present in Maestro 2021 suite [43]. Furthermore, all the missing portions, clashes, and side chains missing in the crystal structure were automatically corrected.

#### 3.4.2. Ligands Preparation

Among the substances found in the tested extract, five were identified as the most abundant phytochemicals present, namely: compound **17** (kaempferol 3-rutinoside-7-rhamnoside), compound **20** (luteolin 7-rutinoside), compound **21** (kaempferitrin), compound **23** (quercetin 3-rhamnoside (quercitrin)) and compound **24** (kaempferol 3-rhamnoside-7-xyloside); they were therefore used to perform the computational experiments including docking and molecular dynamic studies on tyrosinase. The 2D structures of the molecules were downloaded from PubChem Compound Result—NCBI, and prepared by Ligand Preparation tool of [43] by neutralization at pH 7.4 ± 0 by Epik and minimization by the use of OPLS-4 [44].

#### 3.4.3. Molecular Docking

**Figure 6:**
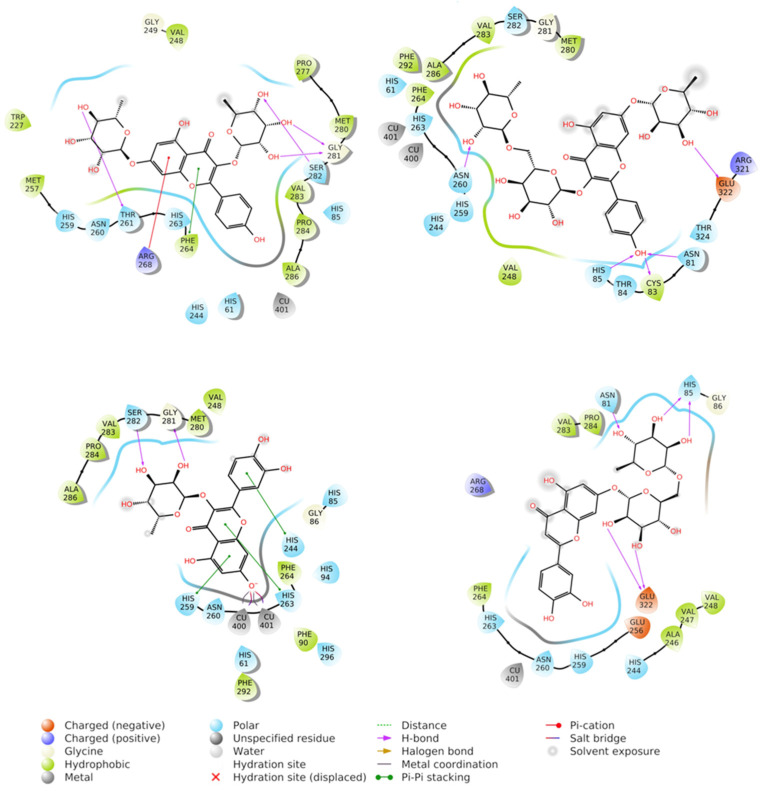
Best docking poses obtained for kaempferitrin (**21**) (upper left), kaempferol 3-rutinoside 7-rhamnoside (**17**) (upper right), quercetin 3-rhamnoside (quercitrin) (**23**) (down left), and luteolin 7-rutinoside (**20**) (down right) docked to tyrosinase.

Glide was employed for the docking to tyrosinase as previously reported by our papers [45,46,47]. In the experiments, the binding pocket was set on the crystallographic ligand, calculating a grid box of 20 Å size. Both Cu atoms contained in tyrosinase have been recognized by the software and will be used for docking experiments. The molecules were first docked by Standard Precision method generating over 300 poses that were then re-docked by eXtra Precision methods, returning the best poses depicted in Figure 6. It should be noted that XP was unable to find a suitable pose for kaempferol 3-rhamnoside-7-xyloside (24), meaning that this compound does not dock well on tyrosinase. Among the XP generated poses, the only one that showed the ligand penetrating the enzymatic pocket and binding to the Cu atoms was quercetin 3-*O*-rhamnoside (quercitrin) (Compound **23** see Table 1). This pose was selected for further studies and subjected to molecular dynamic calculation by the Desmond module implemented in Maestro 2021.

#### 3.4.4. Molecular Dynamic

The best docking scores have been observed for compound **20** and **23**. However, compound **20**, namely luteolin-7-rutinoside was not able to enter in the enzymatic pocket of trosinase and bind derectly to the catalytic Cu atom of the enzyme. On the other hand, compound **23** (quercitrin) penetrated and docked into the enzymatic cavity of tyrosinase thus was selected for further experiment by molecular dynamic calculation using the Desmond module implemented in Maestro 2021 [43,48]. Since this substance was the only one to deeply penetrate the enzymatic poses, the other tested substances were docked only externally to the cavity. In order to study the behavior and goodness of the pose found for compound **23** docked in the enzymatic pocket of tyrosinase along a time scale of nanoseconds, we carried out 10 ns of molecular dynamics simulations (MDS) [49]. TIP3P water molecule standard [50] was employed for the aqueous environment. Orthorhombic periodic boundary parameters were used to build the shape and dimension of the unit buffer at ten angstroms of distance. The charge of the system was neutralized by adding the number of counter ions such as Na^+^/Cl^−^ to neutralize the charges in the system. The ions were randomly inserted by the software in the aqueous environment, and a buffer of 0.1 M of NaCl was also added. After building the aqueous model, the system was minimized to relax the enzyme-inhibitor complex by using the *canonical ensemble* which consists of constant-temperature, constant-volume (NVT), before starting the actual calculation.

MDS were carried out at constant temperature and pressure (NPT). These conditions allow control over both the temperature and pressure using OPLS4 parameters [51,52]. The temperature was set at 309 K and pressure at atmospheric value (1.01 atm) using Nose-Hoover temperature thermostat and isotropic scaling [53].

#### 3.4.5. MD trajectory Analysis

Several parameters have been examined and analyzed in the simulation trajectory. The MDS trajectory file was analyzed by the panel embedded in Desmon module namely “simulation interaction diagram” (SID) in order to calculate the energy, root-mean square fluctuation and deviation (RMSF and RMSD), the stability of the ligand–enzyme bonds, radius of gyration along with secondary structure elements (SSE) of the enzyme which represent the overall stability of the structure [49].

#### 3.4.6. Enzyme Structure Conformational Mobility and Stability Analysis

RMSD for Cα of the tyrosinase and of the ligand 23 docked to tyrosinase has been calculated. RMSD was calculated for the enzyme back-bone in the molecular dynamic simulation starting from the initial structure (Figure 2).

## 4. Conclusions

The results of the present study showed that the leaf of *S. afzelii* exerted significant antioxidant and enzyme inhibition activities and was rich in total phenolics and flavonoid content. The tested biological activities were varied according to the extraction solvent used. Extraction with 80% or 100% methanol recovered biomolecules with the highest antiradicals, total antioxidant, anti-tyrosinase, and anti-α-amylase activities. The former solvent extracted compounds with ion-reducing capacity, anti-cholinesterase, and α-glucosidase inhibitory activities, while the latter solvent showed the best butyrylcholinesterase inhibitory activity. Water as solvent extracted the least amount of total phenolics and flavonoids and consequently revealed the lowest antioxidant and enzyme inhibition activities except its capacity to chelate iron where it showed the highest chelating power. The same was true when increasing the water proportion in methanol (50%) except in its α-glucosidase inhibitory activity where it exerted the same highest activity as the 80% extract. Quantitative analysis revealed that bioactivity is clearly dominated by flavonoids. Extracts were rich in metabolites with abundant accumulation of 2 kaempferol glycosides. Thus, the isolation and characterization of compounds in different active extracts are warranted for further research about their activities and mechanism of action. In that way, this plant species may become an interesting source of bioactive compounds for the food industry, particularly for the development of novel functional foods and/or food supplements.

## Figures and Tables

**Table 1:** Total phenolic and flavonoid content of the tested extracts.

Extracts	Total Phenolic Content (mg GAE/g)	Total Flavanoid Content (mg RE/g)
Methanol (50%)	62.91 ± 1.90 ^b^	17.29 ± 0.15 ^c^
Methanol (80%)	81.69 ± 0.50 ^a^	26.63 ± 0.26 ^b^
Methanol (100%)	82.79 ± 0.90 ^a^	29.96 ± 0.45 ^a^
Water	25.42 ± 0.38 ^c^	11.45 ± 0.06 ^d^

Values are reported as mean ± SD of three parallel measurements. GAE: gallic acid equivalents; RE: rutin equivalents. Different letters indicate significant differences in the tested extracts (*p* < 0.05).

**Table 2:** Characterization of the compounds found in the analyzed extracts of *S. afzelii*.

No.	t*_R_* (min)	[M-H]^−^ *m*/*z*	*m*/*z* (% Base Peak)	Assigned Identification	MeOH	80% MeOH	50% MeOH	H_2_O
**1**	1.8	377	MS^2^ [377]: 341 (100) MS^3^ [377→341]: 179 (100), 161 (18), 143 (22), 131 (9), 113 (19)	Disaccharide (HCl adduct)	✓	✓	✓	
**2**	1.9	191	MS^2^ [191]: 173 (29), 111 (100)	Isocitric acid	✓	✓	✓	✓
**3**	2.7	191	MS^2^ [191]: 173 (24), 111 (100)	Citric acid	✓	✓	✓	✓
**4**	3.7	315	MS^2^ [315]: 153 (100), 109 (14)	Dihydroxybenzoic acid-*O*-hexoside	✓	✓	✓	✓
**5**	4.6	305	MS^2^ [305]: 179 (100) MS^3^ [305→179]: 135 (100)	Caffeic acid derivative	✓	✓	✓	
**6**	5.8	203	MS^2^ [203]: 186 (3), 159 (100), 142 (13) MS^3^ [203→159]: 130 (96), 116 (100)	Tryptophan	✓	✓	✓	✓
**7**	6.0	901	MS^2^ [901]: 781 (69), 739 (100)	Unknown				✓
**8**	6.9	577	MS^2^ [577]: 451 (32), 425 (100), 407 (65), 289 (29), 287 (20)	Procyanidin	✓	✓	✓	
**9**	8.1	577	MS^2^ [577]: 451 (14), 425 (100), 407 (73), 289 (22), 287 (16)	Procyanidin	✓	✓	✓	
**10**	8.8	289	MS^2^ [289]: 245 (100), 205 (37), 203 (25)	Catechin	✓	✓	✓	
**11**	10.7	431	MS^2^ [431]: 385 (100), 223 (13), 153 (9)	Roseoside (formate adduct)	✓	✓	✓	
**12**	12.9	593	MS^2^ [593]: 503 (22), 473 (100), 383 (26), 353 (37)	Apigenin-6,8-di-*C*-glucoside (vicenin-2)	✓	✓	✓	✓
**13**	13.9	593	MS^2^ [593]: 473 (100), 431 (98), 351 (64), 327 (91), 285 (66) MS^3^ [593→431]: 285 (100) MS^4^ [593→431→285]: 255 (100), 151 (7)	Kaempferol-*C*-hexoside-*O*-deoxyhexoside				✓
**14**	15.3	593	MS^2^ [593]: 447 (41), 431 (76), 285 (100) MS^3^ [593→431]: 285 (100) MS^4^ [593→431→285]: 255 (100)	Kaempferol-*O*-hexoside-*O*-deoxyhexoside	✓	✓	✓	✓
**15**	15.4	577	MS^2^ [577]: 451 (14), 425 (100), 407 (43), 289 (36), 287 (23)	Procyanidin	✓	✓	✓	
**16**	16.2	755	MS^2^ [755]: 609 (100), 301 (12) MS^3^ [755→609]: 301 (100) MS^4^ [755→609→301]: 271 (38), 179 (100), 151 (34)	Quercetin-*O*-deoxyhexoside-*O*-rutinoside	✓	✓	✓	✓
**17**	18.3	739	MS^2^ [739]: 593 (100) MS^3^ [739→593]: 285 (100) MS^4^ [739→593→285]: 257 (44), 255 (100), 151 (20)	Kaempferol-3-rutinoside-7-rhamnoside	✓	✓	✓	✓
**18**	19.7	593	MS^2^ [593]: 447 (100), 301 (39) MS^3^ [593→447]: 301 100) MS^4^ [593→447→301]: 271 (100), 255 (20), 179 (5), 151 (88)	Quercetin-*O*-di-deoxyhexoside	✓	✓	✓	✓
**19**	20.0	609	MS^2^ [609]: 301 (100) MS^3^ [609→301]: 271 (57), 179 (52), 151 (100)	Rutin	✓	✓	✓	
**20**	21.9	593	MS^2^ [593]: 285 (100) MS^3^ [593→285]: 255 (100), 243 (29), 241 (16)	Luteolin-7-rutinoside	✓	✓	✓	✓
**21**	22.7	577	MS^2^ [577]: 431 (100) MS^3^ [577→431]: 285 (100) MS^4^ [577→431→285]: 257 (29), 255 (100)	Kaempferitrin (kaempferol-3-7-dirhamnoside)	✓	✓	✓	✓
**22**	23.5	447	MS^2^ [447]: 285 (100) MS^3^ [447→285]: 257 (25), 255 (100), 227 (19)	Kaempferol-*O*-hexoside	✓	✓	✓	✓
**23**	24.8	447	MS^2^ [447]: 301 (100) MS^3^ [447→301]: 271 (20), 179 (79), 151 (100)	Quercitrin (quercetin-3-*O*-rhamnoside)	✓	✓	✓	✓
**24**	25.2	563	MS^2^ [563]: 417 (5), 284 (100) MS^3^ [563→284]: 257 (18), 255 (100)	Kaempferol 3-rhamnoside-7-xyloside	✓	✓	✓	✓
**25**	26.6	781	MS^2^ [781]: 635 (100), 431 (11), 285 (18) MS^3^ [781→635]: 593 (43), 285 (100) MS^4^ [781→635→285]: 257 (100), 243 (14), 241 (51)	Luteolin derivative	✓	✓	✓	✓
**26**	27.6	593	MS^2^ [593]: 285 (100) MS^3^ [593→285]: 243 (100)	Luteolin-*O*-rutinoside	✓	✓	✓	✓

**Table 3:** Quantification of the flavonoids identified in *S. afzelii*.

N°	Assigned Identification	50% MeOH	80% MeOH	MeOH	H_2_O
**12**	Vicenin-2	0.36 ± 0.02 ^a^	0.35 ± 0.02 ^a^	0.22 ± 0.02 ^b^	0.19 ± 0.01 ^b^
**16**	Quercetin-*O*-dHex-*O*-Rut	0.57 ± 0.04 ^ab^	0.65 ± 0.05 ^a^	0.48 ± 0.03 ^b^	---
**17**	Kaempferol-*O*-dHex-*O*-Rut	6.7 ± 0.5 ^a^	5.1 ± 0.4 ^b^	6.4 ± 0.4 ^a^	1.8 ± 0.1 ^c^
**18**	Quercetin-*O*-di-dHex	0.98 ± 0.07 ^b^	1.21 ± 0.08 ^a^	0.78 ± 0.05 ^c^	0.22 ± 0.01 ^d^
**20**	Luteolin-*O*-Rut	1.01 ± 0.07 ^a^	0.66 ± 0.05 ^b^	0.98 ± 0.07 ^a^	0.48 ± 0.03 ^c^
**21**	Kaempferol-*O*-di-dHex	5.4 ± 0.4 ^a^	5.8 ± 0.4 ^a^	5.5 ± 0.4 ^a^	2.0 ± 0.1 ^b^
**23** + **24**	Quercetin + kaempferol Glyc	1.14 ± 0.08 ^a^	1.25 ± 0.09 ^a^	1.26 ± 0.09 ^a^	0.122 ± 0.008 ^b^
**25**	Luteolin derivative	0.31 ± 0.02 ^a^	0.32 ± 0.02 ^a^	0.28 ± 0.02 ^a^	0.18 ± 0.01 ^b^
**26**	Luteolin-*O*-Rut	0.31 ± 0.02 ^a^	0.31 ± 0.02 ^a^	0.27 ± 0.02 ^a^	0.18 ± 0.01 ^b^
Total		16.8 ± 0.7 ^a^	15.7 ± 0.6 ^a^	16.2 ± 0.6 ^a^	5.2 ± 0.2 ^b^

Means in the same line not sharing the same letter are significantly different at *p* < 0.05 probability level, being the letter “a” the highest value. Hex = hexoside; dHex = deoxyhexoside; Rut = rutinoside; Glyc = glycoside.

**Table 4:** Relative peak areas and heat map of extracts of aerial parts of *S. afzelii.* Abbreviations:Hex = hexoside; dHex = deoxyhexoside (mainly rhamnoside); Rut = rutinoside; Pen = pentoside (such as xyloside).

Peak	Compound	50% MeOH	80% MeOH	MeOH	H_2_O
**1**	Disaccharide	1.27	1.63	1.92	0.00
**2**	Isocitric acid	0.75	0.01	0.58	1.57
**3**	Citric acid	0.01	0.01	0.05	0.02
**4**	Dihydroxybenzoic acid-*O*-Hex	0.16	0.36	0.14	0.23
**5**	Caffeic acid derivative	0.32	0.37	0.36	0.00
**6**	Trytophan	0.59	0.66	0.79	0.96
**7**	Unknown	0.00	0.00	0.00	2.39
**8**	Procyanidin	1.00	1.51	1.00	0.00
**9**	Procyanidin	0.65	0.66	0.52	0.00
**10**	Catechin	0.64	0.62	0.61	0.00
**11**	Roseoside	0.64	0.62	1.07	0.00
**12**	Vicenin-2	1.37	1.20	0.64	0.55
**13**	Kaempferol-*C*-Hex-*O*-dHex	0.00	0.00	0.00	4.17
**14**	Kaempferol-*O*-Hex-*O*-dHex	0.98	0.93	0.55	0.75
**15**	Procyanidin	0.42	0.35	0.24	0.00
**16**	Quercetin-*O*-dHex-*O*-Rut	4.40	5.65	3.90	2.13
**17**	Kaempferol-Rut-dHex	32.25	35.31	35.55	38.03
**18**	Quercetin-*O*-di-dHex	5.95	7.82	5.69	5.88
**19**	Rutin	0.39	0.38	0.28	0.00
**20**	Luteolin-*O*-Rut	6.53	4.66	4.43	5.43
**21**	Kaempferol-di-dHex	31.70	27.30	32.44	34.15
**22**	Kaempferol-*O*-Hex	0.09	0.11	0.09	0.10
**23**	Quercitrin	3.89	3.81	3.30	1.46
**24**	Kaempferol-*O*-dHex-*O*-Pen	2.26	2.22	2.25	0.86
**25**	Luteolin derivative	2.45	2.74	2.19	0.91
**26**	Luteolin-*O*-Rut	1.30	1.08	1.39	0.42

**Table 5:** Antioxidant properties of the tested extracts.

Extracts	DPPH (mg TE/g)	ABTS (mg TE/g)	CUPRAC (mg TE/g)	FRAP (mg TE/g)	PBD (mmol TE/g)	MCA (mg EDTAE/g)
Methanol (50%)	48.82 ± 0.05 ^b^	81.70 ± 0.10 ^b^	175.14 ± 3.11 ^c^	108.80 ± 1.03 ^c^	1.63 ± 0.09 ^b^	22.52 ± 1.25 ^b^
Methanol (80%)	248.87 ± 14.23 ^a^	357.90 ± 1.99 ^a^	338.03 ± 7.28 ^a^	215.58 ± 1.61 ^a^	2.16 ± 0.14 ^a^	16.93 ± 1.14 ^c^
Methanol (100%)	266.94 ± 0.40 ^a^	352.39 ± 4.58 ^a^	281.91 ± 10.41 ^b^	204.24 ± 0.67 ^b^	2.01 ± 0.08 ^a^	6.61 ± 0.56 ^d^
Water	30.09 ± 1.30 ^c^	52.59 ± 1.00 ^c^	54.15 ± 1.39 ^d^	37.63 ± 0.42 ^d^	0.85 ± 0.04 ^c^	35.46 ± 0.39 ^a^

Values are reported as mean ± SD of three parallel measurements. PBD: phosphomolybdenum; MCA: metal chelating activity; TE: trolox equivalent; EDTAE: EDTA equivalent. Different letters indicate significant differences in the tested extracts (*p* < 0.05).

**Table 6:** Enzyme inhibitory effects of the tested extracts.

Extracts	AChE (mg GALAE/g)	BChE (mg GALAE/g)	Tyrosinase (mg KAE/g)	Amylase (mmol ACAE/g)	Glucosidase (mmol ACAE/g)
Methanol (50%)	1.97 ± 0.04 ^b^	2.61 ± 0.14 ^b^	63.13 ± 0.58 ^b^	0.49 ± 0.03 ^b^	4.02 ± 0.01 ^a^
Methanol (80%)	2.16 ± 0.10 ^a^	1.96 ± 0.46 ^b^	66.96 ± 0.44 ^a^	0.65 ± 0.01 ^a^	4.01 ± 0.01 ^a^
Methanol (100%)	na	3.50 ± 0.27 ^a^	67.06 ± 1.52 ^a^	0.63 ± 0.01 ^a^	3.87 ± 0.01 ^b^
Water	na	na	na	0.13 ± 0.05 ^c^	0.25 ± 0.03 ^c^

Values are reported as mean ± SD of three parallel measurements. GALAE: galantamine equivalent; KAE: kojic acid equivalent; ACAE: acarbose equivalent; na: not active. Different letters indicate significant differences in the tested extracts (*p* < 0.05).

**Table 7:** Docking values (expressed as glide docking scores, kcal/mol).

Ligand	SP Docking Scores	XP Docking Scores
**23**	−6.372	−9.334
**21**	−6.176	−6.279
**20**	−5.349	−9.394
**24**	−4.528	no pose
**17**	−5.647	−8.556

## Data Availability

Not applicable.

## Sample Availability

Not available.
